# Ultralong persistent luminescence from carbon dots

**DOI:** 10.1038/s41377-022-00818-4

**Published:** 2022-05-10

**Authors:** Seunghyup Yoo, Youngjin Song, Sangin Hahn

**Affiliations:** School of Electrical Engineering, Korea Advanced Institute of Science and Technology (KAIST), Daejeon, 34141 Republic of Korea

**Keywords:** Optical materials and structures, Lasers, LEDs and light sources

## Abstract

Hour-level persistent luminescence is realized with carbon dots embedded in cyanuric acid, the composition of which is easily obtained by the microwave-assisted heating of carbon dots and urea. By forming donor-acceptor blends, the proposed composition yields intermediate states with long lifetimes, providing a rare-earth-metal-free route to ultralong persistent luminescence.

Quantum dots are usually thought of as zero-dimensional nanomaterials based on inorganic compound semiconductors, such as PbS, CdSe, and InP, which are already used in displays for vivid color representation. However, the term “quantum dots” is not limited to these inorganic materials. Since the accidental discovery of fluorescent fragments from single-walled carbon nanotubes in 2004, carbon quantum dots, simply referred to as “carbon dots (CDs),” have been regarded as a new class of nanomaterials that can offer unique light-emitting properties from one of the most abundant materials^1,2^. The environmentally friendly nature, low toxicity^3^, emission tunability^4^, and relatively simple synthetic routes^5^ of CDs have attracted significant interest from a variety of research fields. Consequently, their versatile potential has been demonstrated in applications such as sensing, bioimaging, drug delivery, and displays^1^.

A recent work by Sargent and his coworkers on CD-based deep blue light-emitting diodes, in particular, showed that CDs may not just be promising, but could become an important alternative for established emitter technologies^6^. Side-chain engineering, which reduces thermal vibration and non-radiative pathways, renders luminescence from CDs to exhibit a bright blue emission with a very narrow spectral width that is critical for the development of modern displays requiring a wide color gamut and high dynamic range^6^. It is worth noting that side-chain engineering or surface passivation has been the key to tuning the luminescence properties of CDs since their discovery. This is because radiative recombination from a CD is mostly attributed to recombination with a surface-related origin rather than a bandgap transition from conjugated *π*-domains^1^. Proper surface passivation is considered to enhance the probability of excitons, localized near the edge of a CD unit, recombining radiatively by minimizing exciton quenching.

The work by Jiang et al.^7^ also focuses on interfacial engineering to unlock novel luminescent properties of CDs—ultralong persistent emission that lasts approximately for an hour, which is quite unusual for organic systems, with a few exceptions^8,9^. A mixture of CDs prepared from m-phenylenediamine (m-CDs) and cyanuric acid (CA) forms donor/acceptor blends that yield highly stable intermediate excited states that can slowly recombine radiatively. The excited states responsible for such persistent emission are considered to be an exciplex between m-CD as a donor and CA as an acceptor, which is formed after a sequential process of absorption from an acceptor, charge (hole) transfer, charge separation, and diffusion. At first glance, the process appears to be similar to that of photocarrier generation in solar cells; however, holes, in this case, are localized within m-CDs, unlike in solar cells. As long as an electron in CA approaches the interface with m-CD^+^, it is likely to form an exciplex. This long-lived exciplex radiatively recombines either by itself or after being converted to singlet or triplet excited states of m-CD, producing multi-color emissions according to their respective energies. **(**Fig. 1) It should be noted that excited states with long lifetimes do not always yield efficient afterglow emissions; they must be accompanied by a mechanism or environment that can prevent such excited states from being quenched via oxygen or traps. Formed in situ during a simple synthetic route based on the microwave-assisted heating of m-CDs/urea composition, CA is covalently bonded to m-CD via C-N bonds, providing a robust host environment that can protect exciplex states from being quenched even in ambient air or water.

**Fig. 1:**
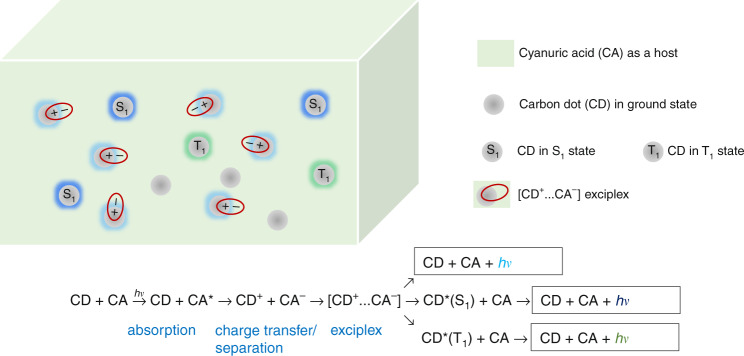
The sequential process leading to formation of exciplex and multi-color long-persistent luminescence (LPL) from carbon dots embedded in cyanuric acid^7^. The color along the rim of a carbon dot in its singlet (S_1_)- and triplet (T_1_) excited states, or exciplex state indicates the approximate color of the corresponding LPL

From a practical perspective, long-persistent luminescence could be useful for glow-in-the-dark paint for emergency signs^7^. Although such an application does not make up a large industry as display or lighting applications, the contributions of the present work can go far beyond. There is considerable room in this nanometric system to discover novel luminescent properties or tune them according to specific needs. Because the mechanism behind light-emitting phenomena from CDs are still being studied, understanding the results from this work may be regarded as an important step for grasping the full picture. As there are many alternatives for host media and substituents, we may be able to identify several other materials that can be paired with carbon dots to further enhance the existing luminescent properties or create new and unusual optoelectronic properties suited for specific needs, as demonstrated in this study.
